# Improving Patient Outcomes by Addressing Provider Variation in Emergency Department Asthma Care

**DOI:** 10.1097/pq9.0000000000000372

**Published:** 2020-12-28

**Authors:** Emily Altick Hartford, Eileen J. Klein, Russell Migita, Stephanie Richling, Jingyang Chen, Lori E. Rutman

**Affiliations:** From the * University of Washington, Seattle Children's Hospital Pediatric Emergency Medicine; † Seattle Children's Hospital.

## Abstract

Asthma exacerbations are frequent in the pediatric emergency department (ED) and result in significant morbidity and costs; standardized treatment improves outcomes. In this study, we aimed to use provider adherence data and the associated patient outcomes as an intervention to change behavior and improve care.

**Methods:**

We used a retrospective cohort design to analyze 2 years of baseline data for asthma patient encounters. Providers were classified based on guideline adherence. We compared patient outcomes by provider adherence using Mann–Whitney U and Fisher’s exact test. Our intervention included education with data feedback and peer comparison. We then analyzed changes in guideline adherence, the proportion of patients admitted, length of stay (LOS), and costs for this population over time using statistical process control charts.

**Results:**

In our baseline data analysis, patients seen by less adherent physicians had a higher likelihood of admission (65.1% versus 50.8%, *P* < 0.001), a longer ED LOS (4.7 versus 4.2 h, *P* = 0.007), and higher costs ($1,896.20 versus $1,728.50, *P* < 0.001). Using SPC analysis, there was an improvement in guideline adherence by providers (64%–77%) with a mirrored improvement in patient adherence (76%–84%) associated with our interventions. Admissions decreased 1 year after the intervention; ED LOS and returns remained unchanged.

**Conclusion:**

In this study, we evaluated patient outcomes according to provider adherence to a clinical guideline and used the results to change provider behavior and improve patient outcomes. Active provision of feedback with peer comparison for providers was associated with improved adherence over time.

## Abstract 2

Supplemental Digital Content is available in the text.

## INTRODUCTION

Asthma is the most common chronic respiratory disease worldwide, and it is the most common chronic disease of childhood.^1^ In the United States, the prevalence of asthma in children is 8.3%, but it is higher among racial minorities and children living below the federal poverty level.^2^ Asthma exacerbation is a common complication, with 53.7% of children with asthma reporting one within the past year.^1^ Children with asthma have significantly higher healthcare utilization and costs than their peers.^3^ The estimated economic burden for pediatric asthma’s direct costs of is 6.31 billion dollars annually in the United States, with a significant portion of this being for the acute management of exacerbations.^3^

Evidence-based national guidelines exist for acute treatment of pediatric asthma exacerbations.^3^ The mainstay of therapy is short-acting bronchodilators, corticosteroids, and supplemental oxygen for hypoxia.^4^ However, there is variation in care across different hospitals, particularly in laboratory evaluation, chest radiography, administration of antibiotics, and the likelihood of admission to the hospital.^5,6^ Standardization of asthma care in the emergency department (ED) and inpatient units via the development of clinical practice guidelines or pathways can improve patient outcomes, including the proportion of hospital admissions, time to medication, length of stay (LOS), and costs.^7,8^

Physician behavior change is increasingly recognized as crucial to the success and sustainability of improvement interventions.^5^ Data audits for physician feedback are widely successful in affecting behavior change, especially for guideline compliance and testing decisions.^6,7^ Data audit and feedback appears to be most efficacious when shared in a timely and frequent manner.^9–11^ Peer comparison to average performance or high performers can also change behavior, and it may be more effective than educational interventions or other process improvements.^12^

**Fig. 1.**
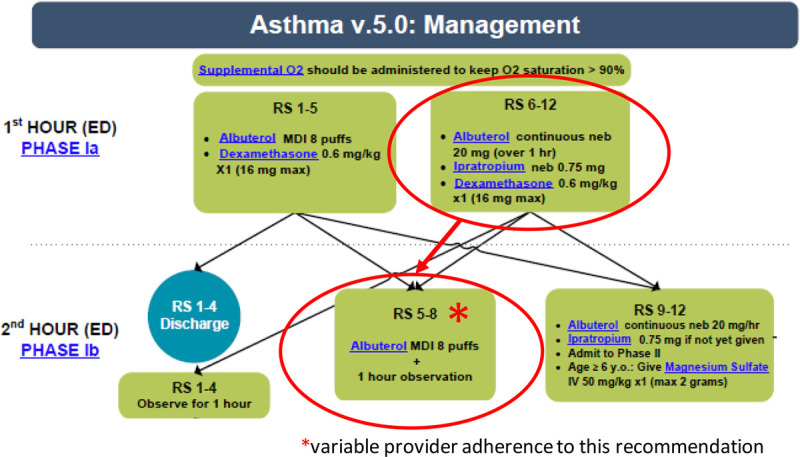
Standardized asthma pathway highlighting the step with variable adherence. *Variable provider adherence to this recommendation.

An evidence-based asthma pathway for the care of children who present with asthma exacerbation was developed at the hospital in 2006. It is reviewed quarterly and updated as necessary with any new or emerging evidence. Overall provider adherence to the asthma pathway is excellent; however, we identified variable adherence to pathway recommendations at a critical step during the second hour of care, which is likely to affect admission decisions, costs, and LOS for patients (Fig. 1). Providers choose between the following options based on the patient’s respiratory score: 1 hour of observation (mild score 1–4), 8 puffs of inhaled albuterol via a metered-dose inhaler and observation (moderate score 5–8), or the second hour of continuous albuterol (20 mg/h) and admission with possible critical care consultation (severe score > 9). We sought to evaluate patient outcomes associated with providers who were adherent and nonadherent, understand the underlying reasons for variation, and develop an intervention to improve provider adherence. Our primary aim was to improve the proportion of patients who receive care according to the pathway by improving provider adherence in the second hour.

## METHODS

### Context

This study took place in a free-standing academic pediatric ED, which receives approximately 50,000 annual visits; 4% of patients have a diagnosis of asthma. Our hospital has a robust system of clinical standardization and guideline development, and providers are accustomed to the following clinical pathways for many common diagnoses. Pathways are updated regularly as new evidence emerges. We have a variety of trainees in our setting who utilize the clinical pathways. Interns and junior residents typically discuss patient care with a fellow or attending physician before initiating treatment; senior residents and fellows have more autonomy in starting patient care before discussion with an attending.

ED staff are generally familiar with quality improvement projects, implementing “plan-do-study-act” cycles, and viewing data on run charts over time. ED attendings have access to various process and outcome metrics on an ongoing basis and are reminded of updates via email monthly with a link. They each have an anonymous code and can view their performance relative to their peers. These metrics are also reviewed annually with the division chief.

### Baseline Data Analysis

**Fig. 2.**
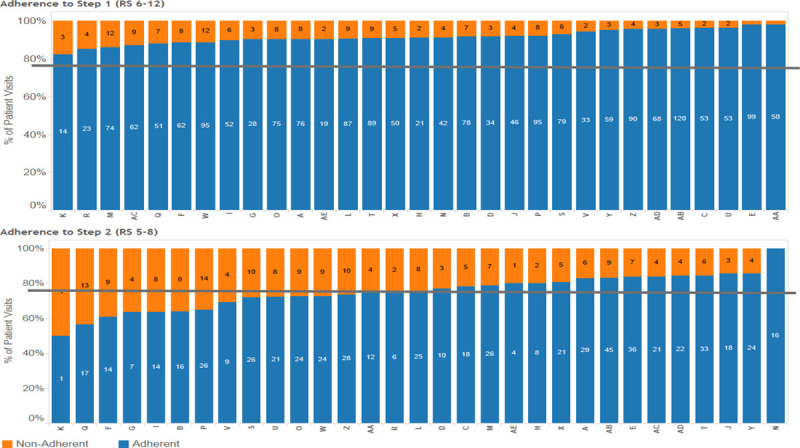
Baseline provider adherence to first- and second-hour pathway recommendations. Blue indicates guideline adherence and orange indicates nonadherence. The gray lines represent a 75% goal; provider adherence is lower at the second hour of care.

We extracted data from the medical record for patients who met the following criteria: presented to the ED and placed on the asthma pathway by the presence of a provider order (the order set includes all pathway care and medications), had a high initial respiratory score (≥6) requiring continuous albuterol, and then had a second respiratory score of moderate severity (5–8) indicating they should then received 8 puffs of albuterol. We analyzed baseline data from July 1, 2013, through August 30, 2015. We evaluated provider adherence to the pathway recommendation for albuterol dosing in the second hour and classified providers as more or less likely to be adherent. We chose this step of the asthma pathway because of provider variation at this step and its significance in determining patient disposition. Deviation from the pathway at the second hour is likely to lead to an admission that might not be necessary. The threshold for more or less adherent providers corresponded to approximately 75% adherence (Fig. 2). Our institutional goal for pathway adherence is typically 80%, instead of 100%, to allow for individual patient variation. We divided providers along the 75% adherence line for practical reasons, as it was close to our institutional goal, and it resulted in equal comparison groups. We then analyzed admissions, ED LOS, and costs between 2 patient cohorts using the exposure variable of provider type, more or less likely to be adherent. Costs were pulled from our hospital accounting system and adjusted for inflation using the US Bureau of Labor Statistics. We included ICU admissions and ED returns within 72 hours as balancing measures. We used Fisher’s exact test and Mann–Whitney U test for comparison of this baseline data.

To explore potential reasons for differences in provider decision-making at this step, we developed an anonymous survey for providers to complete. ED attending physicians completed the survey online after a discussion at a staff meeting in October 2015.

### Intervention

Our improvement intervention included provider education, review of the differences in our baseline data according to provider adherence, and regular provision of data feedback with peer comparison. First, we developed a short and focused educational slide module for providers regarding the differences in patient outcomes associated with pathway adherence in the second hour. Providers completed a mandatory educational intervention over 1 month in November 2015. We reviewed pathway adherence at department operational meetings, by email, and in a weekly newsletter. In December 2015, ED attending physicians started receiving data feedback regarding their adherence to the asthma pathway relative to their colleagues, along with routine monthly metrics. This feedback was then intermittently updated and sent to providers for review.

### Study of the Intervention

We analyzed 5 years of patient data (July 2013–October 2018), including the preintervention and postintervention phases using statistical process control charts (SPC). The population remained the same: patients in the ED placed on the asthma pathway presenting initially with a high respiratory score (≥6) and a moderate respiratory score (5–8) after 1 hour of therapy.

### Measures

We tracked the following outcome measures for the entire timeframe: percent of patients whose care was compliant with the asthma pathway at the second hour, the proportion of patients admitted to the hospital, mean ED LOS, and costs. We also followed the proportion of provider adherence to the asthma pathway during the second hour as a process measure. Balancing measures included ED LOS, unplanned ED return visits within 72 hours for patients discharged from the ED, and any episodes of severe clinical deterioration (defined as transfer to the ICU from the medical floor within 24 h of admission from the ED).

### Analysis

In our baseline data, we compared patient cohorts between more and less adherent providers utilizing the Mann–Whitney U test, a nonparametric test to compare 2 means from the same population, and Fisher’s exact test to determine an association between 2 categorical values.

SPC utilizes Shewhart control charts to evaluate variation in processes over time. Therefore, we used control charts to distinguish variation due to common causes and special assignable causes for our entire study period. We selected chart types based on the data to be analyzed (eg, continuous versus count/classification). A control chart contains a centerline and upper and lower control limits, which are statistically defined, generally 2 SDs above and below the mean. We used standard rules to identify special cause variation.^13^ We utilized QI Charts 2.0 add-on for Microsoft Excel (Process Improvement Products, Austin, Tex.) for control charts.

### Ethical Considerations

The institutional review board determined this study was quality improvement, exempt from a full traditional review.

## RESULTS

### Baseline Provider Adherence

In our baseline data, we found that 15 out of 31 providers were less likely to be adherent at the second hour of care within the asthma pathway as defined by a guideline adherence threshold of 75%. Our baseline data included 742 asthma patient encounters. Patients seen by less adherent providers were more likely to be admitted (65.1% versus 50.8%, *P* < 0.001); had a longer median ED LOS before discharge (4.7 versus 4.2 h, range 2.1–8 versus 2–7 h, *P* = 0.007), and had higher median ED-related costs ($1,896.20 versus $1,728.50, *P* < 0.001) compared to patients seen by more adherent providers. ICU admissions from the ED (1% versus 1.7%, *P* = 0.7), and 72 hour ED returns (4% versus 5.3%, *P* = 0.8) did not differ significantly between the 2 patient groups.

Twenty-five (80.6%) providers completed an anonymous survey; the majority cited patient history of severe asthma, albuterol use before the presentation, and clinical exam as reasons for continuing high-dose continuous albuterol rather than switching to metered-dose inhaler per the pathway recommendations.

### Analysis of the Intervention

**Fig. 3.**
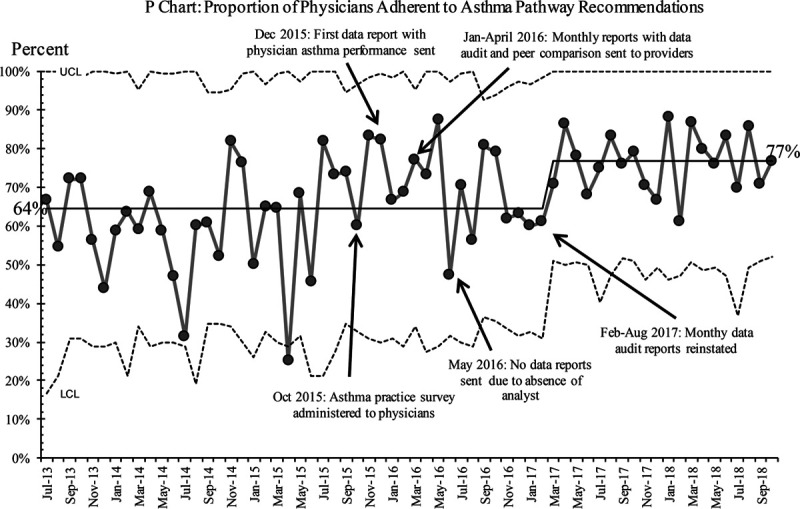
Physician adherence to pathway over time. LCL; UCL.

**Fig. 4.**
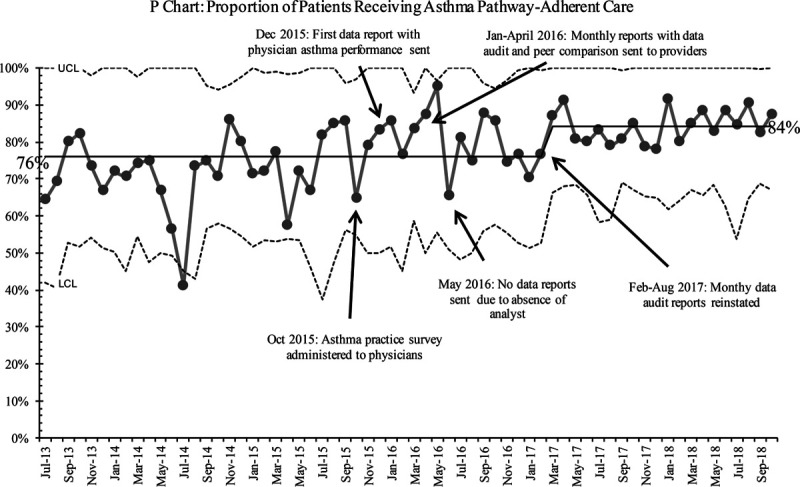
Patients experiencing pathway adherent care over time. LCL; UCL.

**Fig. 5.**
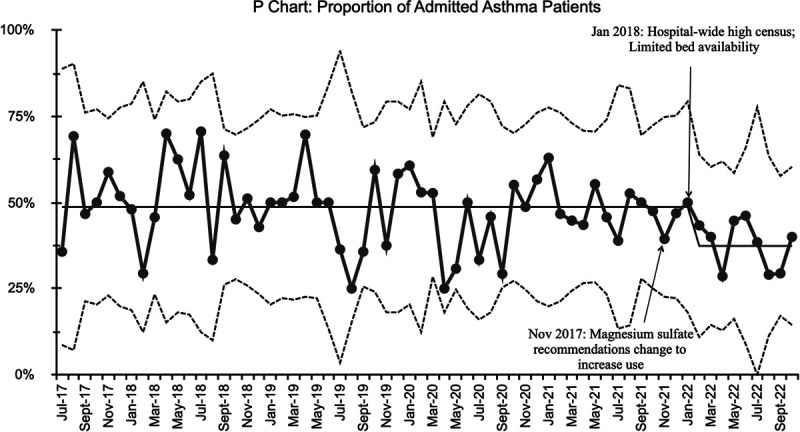
Hospital admissions from the ED for asthma patients in this population.

In the SPC analysis, we noted a shift (8 points above the centerline) toward pathway adherence among physicians after the intervention from 64% to 77% (Fig. 3). The change met special cause variation and was sustained only after the regular and active provision of physician feedback. A higher proportion of patients also received care according to the pathway matching the physician adherence with improvement seen during the same timeframe from 76% to 84% (Fig. 4). The timing of these changes noted by SPC corresponds with the regular provision of feedback via provider metrics with peer comparison. No other interventions were occurring in the same timeframe. Thus, we believe they are related. In our baseline data, we found an association between poorer pathway adherence and a higher likelihood of hospital admission. After the intervention, the proportion of patients admitted to the hospital also decreased. However, this change only met the criteria for special cause variation later in the final year of data monitoring, starting in January 2018 (Fig. 5).

We analyzed ED and hospital costs for this patient population using account information, which was adjusted for inflation and followed throughout the study. These costs remained stable throughout the study period. There was a trend toward lower costs and less variability after our intervention, but they did not meet special cause variation (**see figure 1, Supplemental Digital Content 1,** http://links.lww.com/PQ9/A226).

Balancing measures included ED LOS, unplanned return visits to the ED, and unexpected clinical deterioration with transfer to the ICU. The ED LOS for asthma patients remained unchanged during this period (**see figure 2, Supplemental Digital Content 2,** http://links.lww.com/PQ9/A227). There was also no difference in ED return visits for this population (3.3% versus 3.1%), both for those who went on to be discharged (1.3% versus 1.5%) or admitted (2% versus 1.6%) after their return visit. During the entire study period, including all patient encounters before and after the intervention, there were no patients who experienced severe clinical deterioration.

## DISCUSSION

This study represents a novel method for assessing the impact of provider variation from clinical standards on patient outcomes. In our baseline data, we classified providers as more or less likely to adhere to our asthma pathway and then analyzed patient data in a retrospective cohort design using provider adherence as an exposure. In the baseline analysis, we found significantly worse outcomes for patients seen by less-guideline adherent providers. We presented these data to providers and discussed the implications as an educational intervention. In our discussions with providers, many found the impact on patient outcomes to be quite compelling and motivating to change their behavior. After completing the educational intervention, we then used data audit, feedback, and peer comparison to improve guideline adherence and patient outcomes.

We achieved improvement in our specific aims of improving provider adherence to our clinical guidelines. These improvements occurred after our interventions described above and were sustained after the provision of regular data feedback. We found a decrease in hospital admission for this patient population though this change occurred a year after our interventions. There are a few possible explanations for the timing of this change. In November 2017, we made an additional change to the asthma pathway to give intravenous magnesium sulfate to very severe asthmatics earlier, which can decrease admission rates.^14^ In January 2018, our hospital also experienced limited bed availability, which required the transfer of some admitted patients to other hospitals and may have impacted provider admission decisions. It is possible that the decrease in admissions was related to these factors and not to our interventions, although there was a downward trend before November 2017. During the study period, we found no change in our balancing measures, including ED LOS, ED 72 hour return visits, and unexpected deterioration after admission.

There was still a variation in provider behavior throughout the study period, and we did not achieve perfect guideline adherence. While we believe that perfect adherence is likely not our ideal state, variation from the standard should be driven by individual patients’ unique needs, not provider opinion or deep-rooted practice. A critical aspect of our intervention appears to be the active provision of peer feedback. We were only able to provide regular reminders with the peer comparison data during periods where our department had data analyst support. We annotated the months where providers received these emails as there does seem to be a correlation with adherence as a result. In interventions utilizing data audit, feedback, and/or peer comparison, it may be more useful to send out actively at given intervals for review.

This report demonstrates the potential of clinical standardization as the basis for further improvement. In an ideal state, clinical standardization is merely the first step in an iterative process of learning and improvement. This study shows the potential of using monitoring and feedback regarding adherence to clinical standards to change clinician behavior and improve patient outcomes.

### Limitations

This study has several limitations. Because we looked at aggregate data over time, we were unable to determine a specific characteristic of each patient encounter, which may have affected decision-making. Our data also rely upon the electronic health record, which could have inaccuracies. The respiratory score drives decision-making in our asthma pathway, and there may be variability among nurses or providers who are assigning it. Our baseline data analysis included 2 years of patient data, but some providers had very few patients on the asthma pathway during that timeframe and may have been misclassified. There may be differences in the acuity of patients seen by our physicians. However, nearly all of our ED attending physicians work an equal distribution of shifts, and patients are distributed randomly according to room availability. Because it is randomly assigned, the exposure to patients of differing severity should be equal. Finally, our institution was an early adopter of care standardization via clinical pathways since 2002. Over time, the culture has shifted so that providers are familiar with clinical pathway use and audit and feedback. This infrastructure and level of provider buy-in may limit the generalizability of this intervention to other institutions.

## SUMMARY

In this study, we analyzed 2 years of baseline patient outcome data based on provider adherence to an asthma pathway. We found worse outcomes associated with lower adherence. We then implemented an educational intervention and utilized data audit and feedback with peer comparison to successfully change provider behavior toward guideline adherence and improved patient outcomes. After some initial regression, these improvements were then sustained over the follow-up period.

## DISCLOSURE

The authors have no financial interest to declare in relation to the content of this article.

## Supplementary Material


