# Supplementary figures and images for: Improving Patient Outcomes by Addressing Provider Variation in Emergency Department Asthma Care
Source: Pediatr Qual Saf. 2020 Dec 28;6(1):e372. doi: 10.1097/pq9.0000000000000372 (PMC7774994; doi:10.1097/pq9.0000000000000372)

Supplemental figure 1

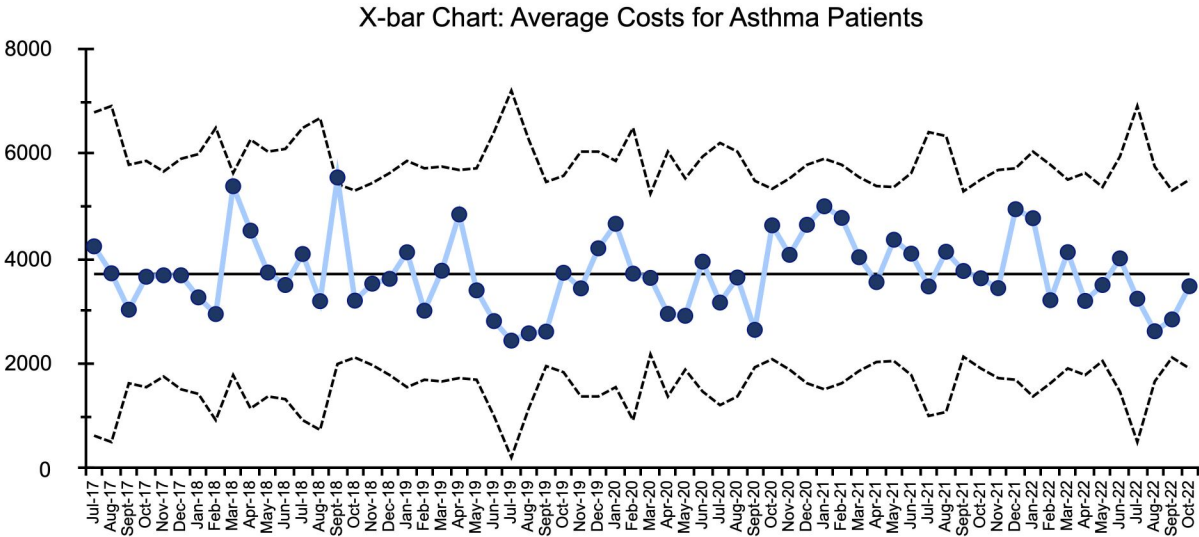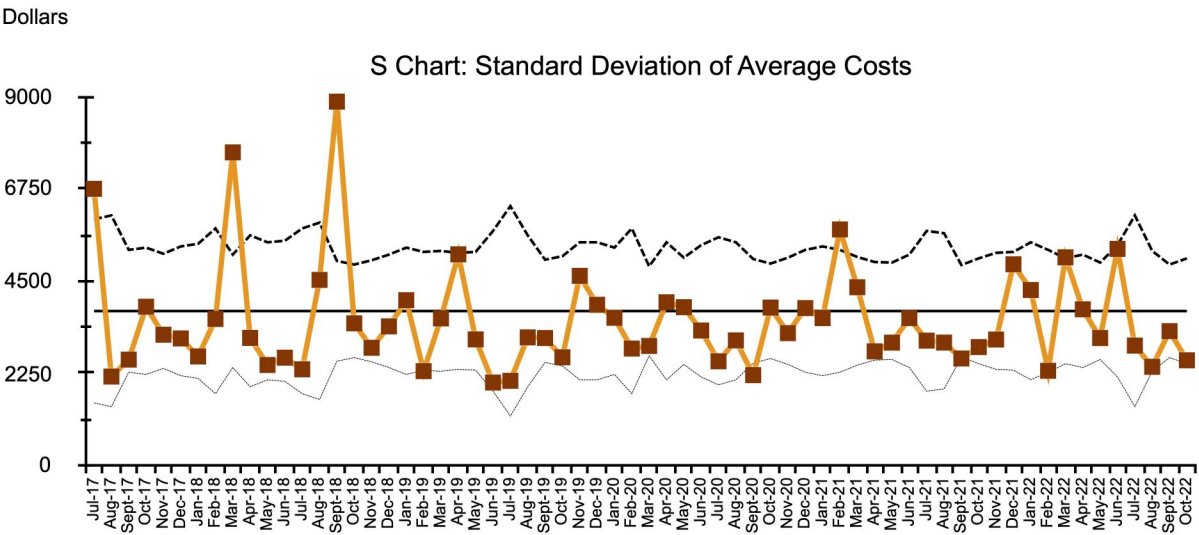

Supplement: Supplementary file 1 [file pqs-6-e372-s001.pdf]

Supplemental figure 2

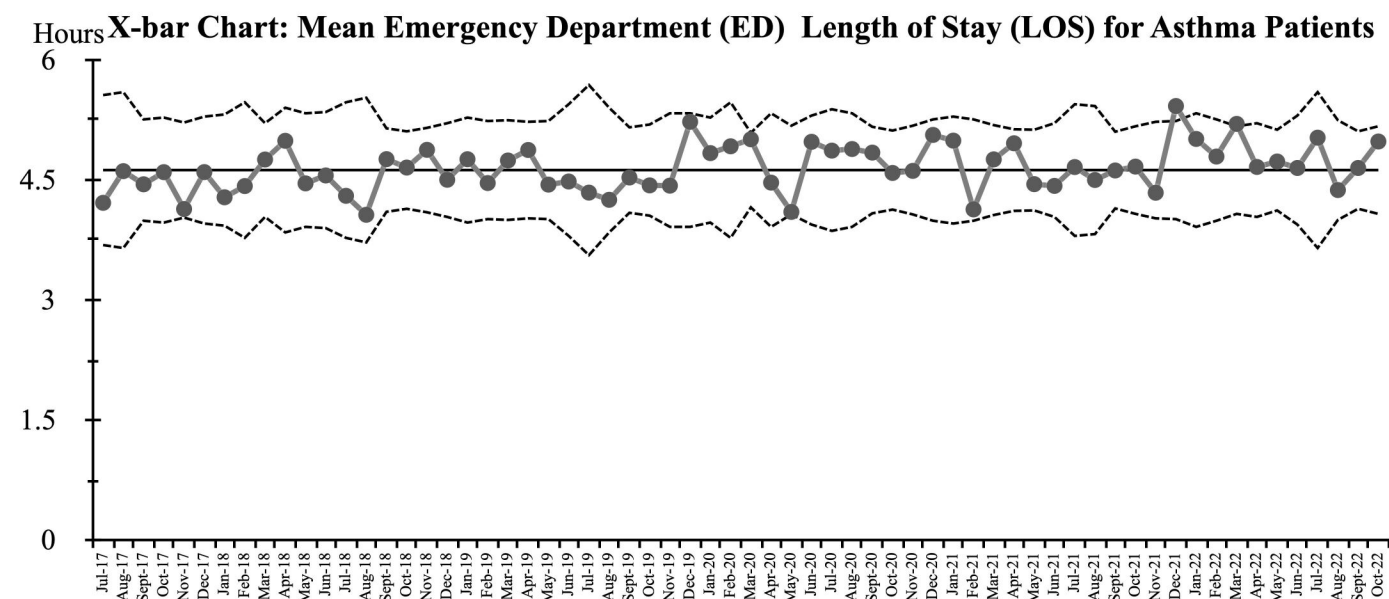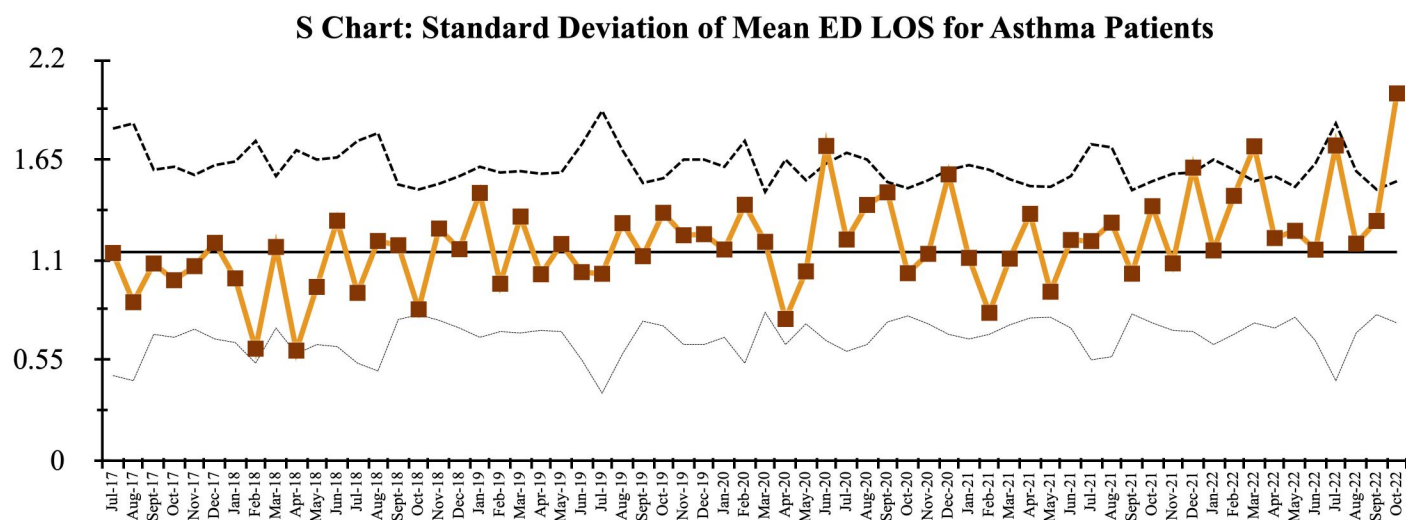

Supplement: Supplementary file 2 [file pqs-6-e372-s002.pdf]
